# Using Si/MoS_2_ Core-Shell Nanopillar Arrays Enhances SERS Signal

**DOI:** 10.3390/nano11030733

**Published:** 2021-03-15

**Authors:** Tsung-Shine Ko, Han-Yuan Liu, Jiann Shieh, De Shieh, Szu-Hung Chen, Yen-Lun Chen, En-Ting Lin

**Affiliations:** 1 Department of Electronic Engineering, National Changhua University of Education, No. 2, Shi-Da Road, Changhua 50074, Taiwan; ihlys1120@gmail.com (H.-Y.L.); shiehjason2@gmail.com (D.S.); seed120x02@gmail.com (Y.-L.C.); ting19960105@gmail.com (E.-T.L.); 2 Department of Materials Science and Engineering, National United University, No. 2, Lianda, Miaoli 36063, Taiwan; jshieh@nuu.edu.tw; 3 Taiwan Semiconductor Research Institute, No. 26, Prosperity Road I, Hsinchu Science Park, Hsinchu 300091, Taiwan; shchen168@narlabs.org.tw

**Keywords:** MoS_2_, Si nanopillar, SERS, core-shell, charge transfer, 2D material

## Abstract

Two-dimensional layered material Molybdenum disulfide (MoS_2_) exhibits a flat surface without dangling bonds and is expected to be a suitable surface-enhanced Raman scattering (SERS) substrate for the detection of organic molecules. However, further fabrication of nanostructures for enhancement of SERS is necessary because of the low detection efficiency of MoS_2_. In this paper, period-distribution Si/MoS_2_ core/shell nanopillar (NP) arrays were fabricated for SERS. The MoS_2_ thin films were formed on the surface of Si NPs by sulfurizing the MoO_3_ thin films coated on the Si NP arrays. Scanning electron microscopy, Raman spectroscopy, and X-ray photoelectron spectroscopy were performed to characterize Si/MoS_2_ core-shell nanostructure. In comparison with a bare Si substrate and MoS_2_ thin film, the use of Si/MoS_2_ core-shell NP arrays as SERS substrates enhances the intensity of each SERS signal peak for Rhodamine 6G (R6G) molecules, and especially exhibits about 75-fold and 7-fold enhancements in the 1361 cm^−1^ peak signal, respectively. We suggest that the Si/MoS_2_ core-shell NP arrays with larger area could absorb more R6G molecules and provide larger interfaces between MoS_2_ and R6G molecules, leading to higher opportunity of charge transfer process and exciton transitions. Therefore, the Si/MoS_2_ core/shell NP arrays could effectively enhance SERS signal and serve as excellent SERS substrates in biomedical detection.

## 1. Introduction

Surface-enhanced Raman scattering (SERS) is a technique for microanalysis and biomedical detection based on the interaction between light and matter [1,2,3,4]. The two widely accepted mechanisms for the SERS effect are the electromagnetic mechanism (EM) and the chemical mechanism (CM). For the EM effect, noble metals are widely used in SERS method because of significant enhancement in signal due to high electromagnetic fields around metallic structures [5,6,7,8]. The presence of metallic structure could provide a boost of several orders of magnitude to SERS signals. On the other hand, the CM effect means the chemical interaction between the analyte molecule and the substrate [9,10,11]. In general, the SERS signal of the CM effect is extremely lower than that of the EM effect. However, using metal substrate has several disadvantages in detecting molecules. For example, the metal catalytic effect would affect the analyte molecule [12], the strong interaction between the metal and the molecule deforms the molecule structure easily [13,14], and the metal surface is prone to produce carbonization effects causing additional strong background signals generated during analysis [15]. In recent years, many studies have demonstrated that 2D materials, such as graphene, boron nitride, and molybdenum disulfide (MoS_2_), could be utilized as SERS substrates for biomedical detections [16,17]. Among the above materials, the MoS_2_ thin film has a flat surface without dangling bonds, which possesses a close proximity to the analyte molecule. Meanwhile, the feature of clean and uniform surface in MoS_2_ is difficult to damage the molecule [18]. Hence MoS_2_ has quite potential in the application of SERS.

To improve the SERS signal of MoS_2_ in detecting the molecule, the fabrication and development of the nanostructure of MoS_2_ for application of biomedical detection is worthy to investigate. Despite that many different types of MoS_2_ nanostructures, such as nanopatterns, nanosheets, and nanospheres, have been reported and proven to be used to enhance photocatalytic reactions by several researchers [19,20,21], little literature is available on the application of MoS_2_ nanostructures to the enhancement of SERS signals. Furthermore, no research has been published regarding Si/MoS_2_ core-shell nanostructure. Since the core-shell nanostructure could affect a larger surface with the analyte molecule during detection [22], the fabrication of a Si/MoS_2_ core-shell nanostructure with a high surface-to-volume ratio is expected to improve the detection signal for the applications of SERS biomedical detection.

In this work, we utilized standard semiconductor process to fabricate periodically distributed Si nanopillars (NPs) with a diameter of about 240 nm over large area. A MoO_3_ layer was deposited on the Si NP arrays using an evaporation system, and then the sample was placed in a high-temperature furnace for sulfurization reaction to form Si/MoS_2_ core-shell NP arrays. We further implemented scanning electronic microscopy (SEM), Raman spectroscopy, and X-ray photoelectron spectroscopy (XPS) to analyze material properties related to the core-shell structure. The Si/MoS_2_ core-shell NP array was used as a SERS substrate to detect the signal of rhodamine 6G (R6G) dye molecules. Raman results show that the SERS signal intensity of R6G molecules detected by the Si/MoS_2_ core-shell NP array can be increased to 75 times that of the Si substrate and 7.5 times that of the MoS_2_ thin film. We will further discuss and explain the possible mechanism in this article. The technique presented here may facilitate the development of MoS_2_ nanostructure. Meanwhile, our results could be able to provide a reference for the use of 2D material nanostructures as SERS substrates in the future.

## 2. Materials and Methods

### 2.1. Fabrication of Ordered Si NP Arrays 

At first, planar p-type Si wafers with resistivity of ~10 ohm-cm were cleaned by a standard wafer clean process. Consequently, we grew thermal oxide with a thickness of ~150 nm as hard mask by using an oxidation system. The as-grown samples were coated by a positive photoresist (PR) layer, exposed to I-line stepper (Canon FPA-3000 i5+) through a mask, and then developed in regular sequence. Afterward, the CF_4_ plasma was applied to etching the exposed area of the SiO_2_ area for 120 s. After the PR layer was removed, we utilized Cl_2_/HBr plasma to etch Si for 250 s and the etch depth was controlled to be about 1000 nm. Eventually, the top oxide hard mask was removed by a plasma asher and buffer oxide etch progress.

### 2.2. Fabrication of Ordered Si/MoS_2_ Core-Shell NP Arrays 

**Figure 1:**
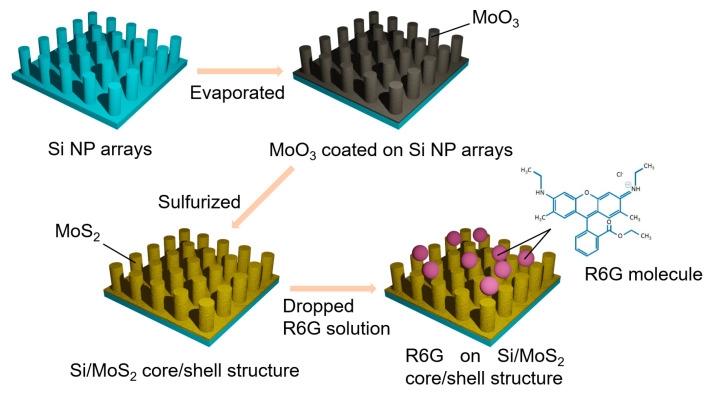
Schematic illustration of the fabrication of Si/MoS_2_ core/shell NP arrays.

In order to understand the effect of using Si NP arrays for SERS, we prepared both bare Si and Si NP arrays as MoS_2_ substrates for comparison. We adopted the same growth procedure of MoS_2_ for both substrates as the following. The schematic of flowchart is shown in Figure 1. The synthesis procedures of crystal MoS_2_ started with MoO_3_ thin film coated on the Si substrates by an evaporator. Both Si and Si NP arrays substrates were pasted on the ceiling of evaporator and faced downward a tungsten boat inside the chamber. A 500 mg MoO_3_ tablet was placed on the tungsten boat and heated around 1000 °C for 30 s. Chamber pressure is about 5 × 10^−5^ torr. Then the samples were sent to a two-zone vacuum furnace for further sulfurization process to grow MoS_2._ The samples were fixed and inserted into a customized quartz plate with 3 mm depth grooves and faced to upstream to ensure MoO_3_ coated on the cylindrical sides of the Si NP arrays could fully sulfurized during the process. In the two-zone vacuum furnace heated with two different temperatures, the 8 g sulfur powders were placed at the upstream source zone while the both kinds of substrates were placed at the downstream reaction zone. We only kept the temperatures at 700 °C for 300 s in the reaction zone. The temperature in source zone reached about 500 °C when the temperature in reaction zone was 700 °C. The temperature ramping rate was set to be 5 °C /min for heating and cooling procedures. The argon with fixed flow of 200 sccm was used as the carrier gas and the pressure was around 0.1 torr. The source vapor was transported from the source zone by the carrier gas and condensed on the substrates in the reaction zone to trigger a sulfurization reaction.

### 2.3. Preparation of R6G Molecules 

R6G dye (ACROS Organics, 99%) was dissolved in water (1 mM and 10 mM) and was dip-coated onto substrates including Si, MoS_2_ thin films on Si, and Si/MoS_2_ core-shell NP arrays. The substrates were then dried by a hot plate with setting 70 °C.

### 2.4. Characterizations 

SEM (FEI Helios 1200+) was used for investigation of the morphology and nanostructure of the as-grown samples. The chemical configurations of the samples related to MoS_2_ were determined by X-ray photoelectron spectroscope (ULVAC-PHI Phi V5000), performed with an Al Kα X-ray source on the samples. The Raman spectra and SERS analyses were recorded from a confocal Raman microscopy system (Tokyo Instruments, Inc., Nanofinder 30) with a He-Ne laser excitation (laser power: 0.05 mW). The laser beam was focused in a spot of 1 μm diameter by a microscope objective with a magnification of 100 X and NA of 0.9. The acquisition time was set to 20 s for the laser measurement. The grating in spectrometer is 300 line/mm. A charge coupled device mounted in the spectrometer was cooled down about -50 °C by using a thermoelectric cooling chip to reduce noise during measurement. The spectra were calibrated by Si peak position (520 cm^−1^) from bulk Si substrate.

## 3. Results and Discussion

**Figure 2:**
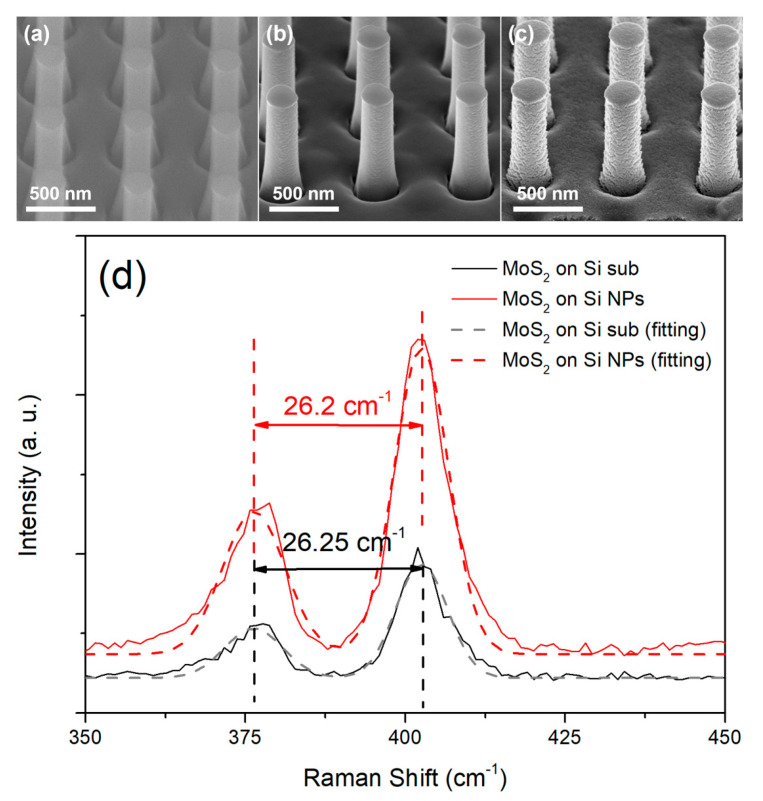
SEM images of (**a**) Si NP arrays, (**b)** MoO_3_ thin films deposited on Si NP arrays, and (**c**) Si/MoS_2_ core/shell NP arrays. (**d**) Raman spectra and corresponding Gaussian fitting results of planar MoS_2_ thin film and Si/MoS_2_ core/shell NP arrays.

Figure 2a–c shows the tilt SEM images of the Si NP arrays before and after deposition of MoO_3_ as well as the Si/MoS_2_ core-shell NP arrays after sulfurization progress. From Figure 2a we observed that the diameter of a Si NP is about 240 nm. After MoO_3_ was deposited, the diameter of the NP is slightly increased to about 255 nm, as shown in Figure 2b. Subsequently, we found that the diameter of the NP is about 287 nm when the outer MoO_3_ was transformed into MoS_2_ and formed Si/MoS_2_ core-shell NP structure after sulfurization, as shown in Figure 2c. From above SEM results, we could estimate that the thickness of the MoO_3_ layer is about 7–8 nm, while the thickness of the MoS_2_ is about 23 nm formed on the lateral sides of Si NP. Note that the thickness of the MoS_2_ is about 3–4 times thicker from the conversion of the MoO_3_ layer, which is similar to the previous studies [23,24]. Moreover, we analyzed the molecule vibration modes for the planar MoS_2_ formed on Si and Si/MoS_2_ core-shell NP arrays substrates under the same MoS_2_ growth conditions using Raman spectroscopy. The Raman spectra and corresponding Gaussian fitting results for both planar MoS_2_ and Si/MoS_2_ core-shell NP arrays are shown in Figure 2d, which show that both samples exhibit E^1^_2g_ and A^1^_g_ typical vibration mode peaks of MoS_2_, suggesting the MoO_3_ layer had been successfully sulfurized into MoS_2_. It is well known that the difference in wavenumber between the two modes would decrease with decreasing thickness of MoS_2_ from bulk to a few monolayers [25,26]. Since our Gaussian fitting results reveal that the difference in wavenumber between the two peaks is about 26.25 cm^−1^ for the planar MoS_2_ and 26.2 cm^−1^ for the Si/MoS_2_ core-shell NP arrays, we could further confirm that the thickness of the MoS_2_ thin film formed on the Si NP is bulk, which is consistent with above SEM observation. 

**Figure 3:**
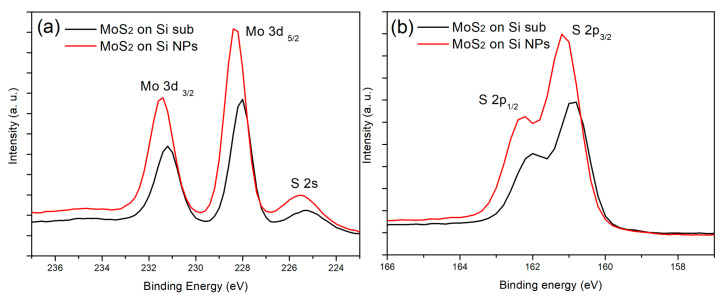
Mo 3d (**a**) and S 2p (**b**) core level XPS spectra of planar MoS_2_ thin film and Si/MoS_2_ core/shell NP arrays.

Figure 3 shows the XPS results of the planar MoS_2_ and the Si/MoS_2_ core-shell NP arrays. The chemical composition and structural information can be further confirmed through bonding energy analysis. In Figure 3a, both XPS spectra of the planar MoS_2_ and the Si/MoS_2_ core-shell NP arrays display Mo 3d_3/2_, 3d_5/_, and S 2S located at the energy positions of 231.6, 228.5, and 225.7 eV. Figure 3b shows that S 2p_1/2_ and S 2p_3/2_ are located near the energy of 162.2 and 161.2 eV, respectively. These peak positions are in good agreement with the results of previous relevant reports, which also determine the MoS_2_ is in 1T stacked structure in this study [27,28,29]. On the other hand, all peaks of the planar MoS_2_ are slightly lower than that of Si/MoS_2_ core-shell NP arrays. Mahatha et al. found that the more dislocation defects and higher surface step density in MoS_2_ structure would reduce the relevant bonding energies of Mo and S atoms [30]. Based on this result, we infer that the use of Si NP arrays could effectively reduce defects. In addition, our XPS results did not show relevant bonding energy related to MoO_3_ [31], indicating that the MoO_3_ layer has fully reacted with sulfur vapor and transferred into MoS_2_ on either on the planar Si or the Si NP arrays after the sulfurization process. 

**Figure 4:**
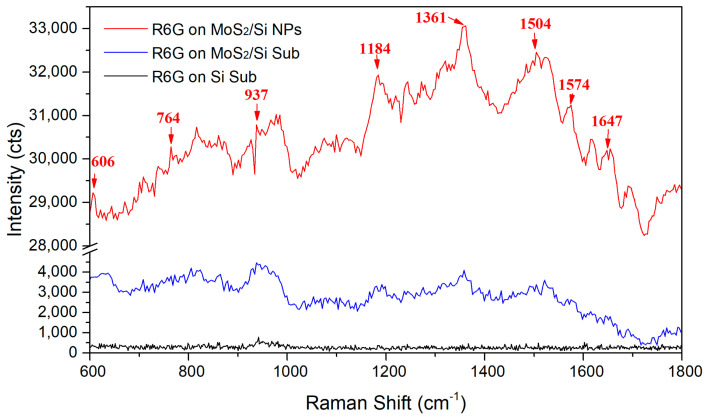
Surface-enhanced Raman scattering (SERS) signals of the R6G molecules (1 mM) on the Si, MoS_2_ thin film, and Si/MoS_2_ core-shell NP arrays substrates.

We selected R6G molecules (1 mM) for the Si substrate, MoS_2_ thin film, and Si/MoS_2_ core-shell NP arrays to compare the sensitivity of SERS application. Figure 4 shows the SERS signals of the substrates obtained by Raman spectroscopy for R6G detection. It is obvious that the Raman intensity is significantly enhanced when R6G molecules are covered on the Si/MoS_2_ core-shell NP arrays. The intensity of all molecular vibration modes related to R6G is much higher than that of covering the MoS_2_ thin film and Si substrate [32]. The peak intensity of using the Si/MoS_2_ core-shell NP arrays is 7.5 times that of using the MoS_2_ thin film and 75 times that of using the Si substrate for the peak 1361 cm^−1^. The surface area ratio of the two substrates Si/MoS_2_ core-shell NP arrays and MoS_2_ was estimated to be about 5.22 based on the SEM observation. Therefore, the higher enhancement of the SERS signal is related to the higher surface-to-volume ratio of the Si/MoS_2_ core-shell NP arrays. It is worth mentioning that the Si NP array is a superhydrophilic substrate [33]. This superhydrophilic characteristic would cause the dropped R6G molecular solution to spread rapidly. In general, the larger spread R6G solution area leads to lower surface distribution density of R6G molecules. Instead, the NP arrays structure additionally provides nearly 5 times area comparing to the planar thin film, making it difficult for the R6G molecules to stack with each other. As a result, more R6G molecules could directly attach to the MoS_2_ thin film on Si NP and thus improve SERS signals. 

In addition to R6G molecule vibration modes, the SERS result of the Si/MoS_2_ core-shell NP arrays also exhibit a broad band feature. Since whole measurements were carried out on almost the same period and under the same conditions, we believe that the broad band of SERS signal for the R6G on the Si/MoS_2_ NP arrays could not originated from relevant R6G molecule vibrations. Instead, because we used the He-Ne laser (~633 nm) as excitation source for SERS measurement, the wavenumber range 600–1800 cm^−1^ could be converted to the corresponding wavelength range 658–714 nm, which is very close to R6G original red-orange color. Therefore, we could suggest that the original color of R6G caused the broad band feature, which would not conflict with the idea that the Si/MoS_2_ core/shell NP arrays with larger area could absorb more R6G molecules. In spite of the board band feature, we still could distinguish molecule vibration modes of R6G labeled using arrow marks from the SERS signals of using the Si/MoS_2_ core/shell NP arrays in Figure 4. The intensities of these modes by using the Si/MoS_2_ core/shell NP arrays are actually stronger than that using either the planar MoS_2_ thin film or the Si substrate. In order to confirm the reproducibility and accuracy for the SERS result, we measured additional SERS signals from the different positions of the Si/MoS_2_ core/shell NP arrays, which is shown in Figure S1a–d of Supplementary Materials. We also measured an additional SERS signal of the R6G molecules (10 mM) on a Si substrate for comparison. Even though the random distribution of R6G molecules introduce the different SERS profiles, obviously the SERS results at the different positions indicate that the enhancement of the Si/MoS_2_ core-shell NP arrays as SERS substrates for R6G molecules is actually stronger than that of the planar MoS_2_ thin film and the Si substrate. Meanwhile, the results of the vibration modes are consistent with the spectrum of the R6G (10 mM) on the Si substrate. Figure S1d shows a relative low intensity in comparison to Figure S1a–c, probably due to low R6G concentration at the position where the drop point of the R6G droplet is far away.

**Figure 5:**
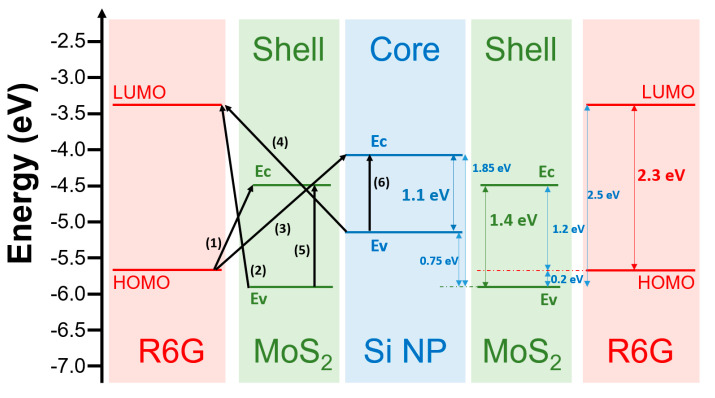
Band diagram schematic of charge transfer (CT) process between R6G molecule and Si/MoS_2_ core-shell NP.

To date, many studies have demonstrated that the SERS mechanism of using 2D semiconductor material is CM and mainly due to charge transfer (CT) process happening on the interface between the molecule and the semiconductor [34,35,36,37]. There are two CT paths between molecule and semiconductor. One is that the electrons in the highest occupied molecular orbital (HOMO) of the molecules absorb enough light energy and then jump to the adjacent semiconductor conduction band to form free electrons, leaving a hole in the HOMO and forming an electron-hole pair, so called exciton. The other is that the electrons in the valence band of the semiconductor transit to the lowest unoccupied molecular orbital (LUMO) of the molecules. A schematic band diagram of the CT process between the R6G and the Si/MoS_2_ core-shell NP arrays is presented in Figure 5. The figure suggested that the electrons excited were transferred to a higher energy state upon absorption of light with a photon energy exceeding the energy differences via the possible transition paths (1)–(6), including (1) the HOMO of R6G to the conduction band of MoS_2_, (2) the valence band of MoS_2_ to the LUMO of R6G, (3) the HOMO of R6G to conduction band of the Si NP, (4) the valence band of Si NP to the LOMO of R6G, (5) the indirect energy gap of MoS_2_, and (6) the indirect energy gap of the Si NP. The corresponding energy difference is (1) 1.2 eV, (2) 2.5 eV, (3) 1.65 eV, (4) 1.75 eV, (5) 1.4 eV, and (6) 1.1 eV, respectively. Since the energy difference in path (2) is about 2.5 eV, which is larger than the photo energy 1.96 eV of the excited laser (~633 nm) we used in this study, the transition through path (2) could not happen. We believe that the opportunities of the transition path (3) and (4) are extremely low because the electrons excited via the paths have to spend much energy to pass through the thickness of MoS_2_ except for the R6G molecule that directly contacts the Si NP through the cracks on the surface of MoS_2_. Furthermore, the laser energy could penetrate either the R6G or MoS_2_ layers to excite electrons in the MoS_2_ thin film and Si NP during the SERS measurement process, leading to the path (5) and (6) related to the internal transition of indirect band gap. Therefore, the main reasons for the SERS enhancement mechanism of the Si/MoS_2_ core-shell NP arrays in the detection of R6G molecules may be attributed to CT process of path (1) and the exciton transitions of path (5) and (6). In comparison with the planar MoS_2_ thin film, we suggest that the Si/MoS_2_ core-shell NP arrays with higher surface-to-volume ratio could absorb more R6G molecules effectively and increase larger interface area between MoS_2_ and R6G. The larger interface area would further promote higher opportunity in CT and exciton resonance, leading to the enhancements R6G signals in SERS analysis. In the future, we would arrange systematically further optical experiments based on this result to understand the contribution and proportion in the SERS enhancement in terms of CT process and exciton transitions.

## 4. Conclusions

In conclusion, we fabricated the Si NP arrays with a diameter of about 240 nm by using the standard semiconductor process. A MoO_3_ layer was deposited on the Si NP arrays in an evaporation system and placed in a high-temperature furnace tube for sulfurization. Raman and XPS results confirm that the MoO_3_ layer around the Si NP completely transferred into MoS_2_ layer with a thickness of 23 nm and formed the Si/MoS_2_ core-shell NP arrays structure. Raman results show that using the Si/MoS_2_ core-shell NP arrays as a SERS substrate could enhance the R6G signals. In comparison with the planar MoS_2_ thin film, the superhydrophilic Si/MoS_2_ core-shell NP arrays with larger area could not only absorb more R6G molecules, but also cause R6G molecules difficult to stack with each other. Hence the core-shell NP arrays provide larger interface area between R6G and MoS_2_, leading higher opportunity of CT process and exciton transitions. The effect could enhance the SERS signals of R6G molecules. Our work indicates that the Si/MoS_2_ core-shell NP arrays is an excellent SERS substrate with great potential. Meanwhile, we believe this discovery will expand the application of 2D material core-shell nanostructure to the biomedical detection.

## Data Availability

Data is contained within the article or Supplementary Material.

## Supplementary Materials

The following are available online at https://www.mdpi.com/2079-4991/11/3/733/s1, Figure S1. SERS signals of the R6G molecules on the Si, MoS2 thin film and Si/MoS2 core-shell NP arrays substrates. An additional SERS signal of the R6G molecules (10 mM) on a Si substrate is included for comparison. The red SERS curves in (a)–(d) were measured from the different positons of the R6G molecules on the Si/MoS2 core-shell NP arrays substrates.

## References

[1] Wu D.-Y., Liu X.-M., Duan S., Xu X., Ren B., Lin S.-H., Tian Z.-Q. (2008). Chemical Enhancement Effects in SERS Spectra:  A Quantum Chemical Study of Pyridine Interacting with Copper, Silver, Gold and Platinum Metals. J. Phys. Chem. C.

[2] Yamada H., Nagata H., Toba K., Nakao Y. (1987). Charge-transfer band and sers mechanism for the pyridine-Ag system. Surf. Sci..

[3] Mosier-Boss P.A. (2017). Review of SERS Substrates for Chemical Sensing. Nanomaterials.

[4] Schatz G.C., Young M.A., Duyne R.P.V., Kneipp K., Moskovits M., Kneipp H. (2006). Topics in Applied Physics. Surface-Enhanced in Applied Physics.

[5] Freeman R.G., Grabar K.C., Allison K.J., Bright R.M., Davis J.A., Guthrie A.P., Hommer M.B., Jackson M.A., Smith P.C., Walter D.G. (1995). Self-Assembled Metal Colloid Monolayers: An Approach to SERS Substrates. Science.

[6] Singh J.P., Chu H.Y., Abell J., Tripp R.A., Zhao Y. (2012). Flexible and mechanical strain resistant large area SERS active substrates. Nanoscale.

[7] Abdelsalam M.E., Bartlett P.N., Baumberg J.J., Cintra S., Kelf T.A., Russell A.E. (2005). Electrochemical SERS at a structured gold surface. Electrochem. Commun..

[8] Dridi H., Haji L., Moadhen A. (2017). Studies of SERS efficiency of gold coated porous silicon formed on rough silicon backside. Appl. Surf. Sci..

[9] Xia L., Chen M., Zhao X., Zhang Z., Xia J., Xu H., Sun M. (2014). Visualized method of chemical enhancement mechanism on SERS and TERS. J. Raman Spectrosc..

[10] Chen R., Jensen L. (2020). Interpreting the chemical mechanism in SERS using a Raman bond model. J. Chem. Phys..

[11] Sharma B., Frontiera R.R., Henry A.-I., Ringe E., Duyne R.P.V. (2012). SERS: Materials, applications, and the future. Mater. Today.

[12] Hvolbæk B., Janssens T.V.W., Clausen B.S., Falsig H., Christensen C.H., Nørskov J.K. (2007). Catalytic Activity of Au nanoparticles. Nano Today.

[13] Guerrini L., Jurasekova Z., Domingo C., Pérez-Méndez M., Leyton P., Campos-Vallette M., Garcia-Ramos J.V., Sanchez-Cortes S. (2007). Importance of Metal–Adsorbate Interactions for the Surface-enhanced Raman Scattering of Molecules Adsorbed on Plasmonic Nanoparticles. Plasmonic.

[14] Dery S., Berg I., Kim S., Cossaro A., Verdini A., Floreano L., Toste F.D., Gross E. (2020). Strong Metal–Adsorbate Interactions Increase the Reactivity and Decrease the Orientational Order of OH-Functionalized N-Heterocyclic Carbene Monolayers. Langmuir.

[15] Xu W., Ling X., Xiao J., Dresselhaus M.S., Kong J., Xu H., Liu Z., Zhang J. (2012). Surface enhanced Raman spectroscopy on a flat graphene surface. Proc. Natl. Acad. Sci. USA.

[16] Qiu H., Li Z., Gao S., Chen P., Zhang C., Jiang S., Li H. (2015). Large-area MoS_2_ thin layers directly synthesized on Pyramid-Si substrate for surface-enhanced Raman scattering. RSC Adv..

[17] Dai Z., Xiao X., Wu W., Zhang Y., Liao L., Guo S., Jiang C. (2015). Plasmon-driven reaction controlled by the number of graphene layers and localized surface plasmon distribution during optical excitation. Light-Sci. Appl..

[18] Ling X., Fang W.J., Lee Y.H., Araujo P.T., Zhang X., Rodriguez-Nieva J.F., Lin Y.X., Zhang J., Kong J., Dresselhaus M.S. (2014). Raman Enhancement Effect on Two-Dimensional Layered Materials: Graphene, h-BN and MoS_2_. Nano Lett..

[19] Zhang X., Suo H., Zhang R., Niu S., Zhao X.Q., Zheng J., Guo C. (2018). Photocatalytic activity of 3D flower-like MoS_2_ hemispheres. Mater. Res. Bull..

[20] Vattikuti S.V.P., Byon C., Reddy C.V., Ravikumar R.V.S.S.N. (2015). Improved photocatalytic activity of MoS_2_ nanosheets decorated with SnO_2_ nanoparticles. RSC Adv..

[21] Liu Y., Hu K., Hu E., Guo J., Han C., Hu X. (2017). Double hollow MoS_2_ nano-spheres: Synthesis, tribological properties, and functional conversion from lubrication to photocatalysis. Appl. Surf. Sci..

[22] Gu L., Zheng K., Zhou Y., Li J., Mo X., Patzke G.R., Chen G. (2011). Humidity sensors based on ZnO/TiO_2_ core/shell nanorod arrays with enhanced sensitivity. Sens. Actuators B Chem..

[23] Heyne M.H., Chiappe D., Meersschaut J., Nuytten T., Conard T., Bender H., Huyghebaert C., Radu I.P., Caymax M., Marneffe J.-F.D. (2016). Multilayer MoS_2_ growth by metal and metal oxide sulfurization. J. Mater. Chem. C.

[24] Kumar P., Singh M., Sharma R.K., Reddy G.B. (2015). Sulfurization of α-MoO_3_ nanostructured thin film. AIP Conf. Proc..

[25] Lee C., Yan H., Brus L.E., Heinz T.F., Hone J., Ryu S. (2010). Anomalous Lattice Vibrations of Single- and Few-Layer MoS_2_. ACS Nano.

[26] Li H., Yin Z., He Q., Li H., Huang X., Lu G., Fam D.W.H., Tok A.I.Y., Zhang Q., Zhang H. (2012). Fabrication of Single- and Multilayer MoS_2_ Film-Based Field-Effect Transistors for Sensing NO at Room Temperature. Small.

[27] Chen M., Ji B., Dai Z., Du X., He B., Chen G., Liu D., Chen S., Lo K.H., Wang S. (2020). Vertically-aligned 1T/2H-MS_2_ (M = Mo, W) nanosheets for surface-enhanced Raman scattering with long-term stability and large-scale uniformity. Appl. Sur. Sci..

[28] Zhang C., Wang Z., Bhoyate S., Morey T., Neria B.L., Vasiraju V., Gupta G., Palchoudhury S., Kahol P.K., Mishra S.R. (2017). MoS_2_ Decorated Carbon Nanofibers as Efficient and Durable Electrocatalyst for Hydrogen Evolution Reaction. C J. Carbon Res..

[29] Vangelista S., Cinquanta E., Martella C., Alia M., Longo M., Lamperti A., Mantovan R., Basset F.B., Pezzoli F., Molle A. (2016). Towards a uniform and large-scale deposition of MoS_2_ nanosheets via sulfurization of ultra-thin Mo-based solid films. Nanotechnology.

[30] Mahatha S.K., Menon K.S.R. (2012). Inhomogeneous band bending on MoS_2_ (0001) arising from surface steps and dislocations. J. Phys. Condens. Matter.

[31] Torres J., Alfonso J.E., López-Carreño L.D. (2005). XPS and X-ray diffraction characterization of MoO_3_ thin films prepared by laser evaporation. Phys. Stat. Sol. (C).

[32] Bai S., Serien D., Hu A., Sugioka K. (2018). 3D microfluidic surface-enhanced Raman spectroscopy (SERS) chips fabricated by all-femtosecond-laser-processing for real-time sensing of toxic substances. Adv. Funct. Mater..

[33] Fan J.-G., Tang X.-J., Zhao Y.-P. (2004). Water contact angles of vertically aligned Si nanorod arrays. Nanotechnology.

[34] Lombardi J.R., Birke R.L. (2014). Theory of Surface-Enhanced Raman Scattering in Semiconductors. J. Phys. Chem. C.

[35] Lee Y., Kim H., Lee J., Yu S.H., Hwang E., Lee C., Ahn J.H., Cho J.H. (2016). Enhanced Raman Scattering of Rhodamine 6G Films on Two-Dimensional Transition Metal Dichalcogenides Correlated to Photoinduced Charge Transfer. Chem. Mater..

[36] Muehlethaler C., Considine C.R., Menon V., Line W.-C., Lee Y.-H., Lombardi J.R. (2016). Ultrahigh Raman Enhancement on Monolayer MoS_2_. ACS Photonics.

[37] Kitadai H., Wang X., Mao N., Huang S., Ling X. (2019). Enhanced Raman Scattering on Nine 2D van der Waals Materials. J. Phys. Chem. Lett..

