# Supplementary material for: Using Si/MoS2 Core-Shell Nanopillar Arrays Enhances SERS Signal
Source: Nanomaterials (Basel). 2021 Mar 15;11(3):733. doi: 10.3390/nano11030733 (PMC8001147; doi:10.3390/nano11030733)
Supplement: Supplementary file 1 [file nanomaterials-11-00733-s001.pdf]

nanomaterials

MDPI

Supplementary materials

Using Si/MoS2 core-shell nanopillar arrays enhances SERS Signal

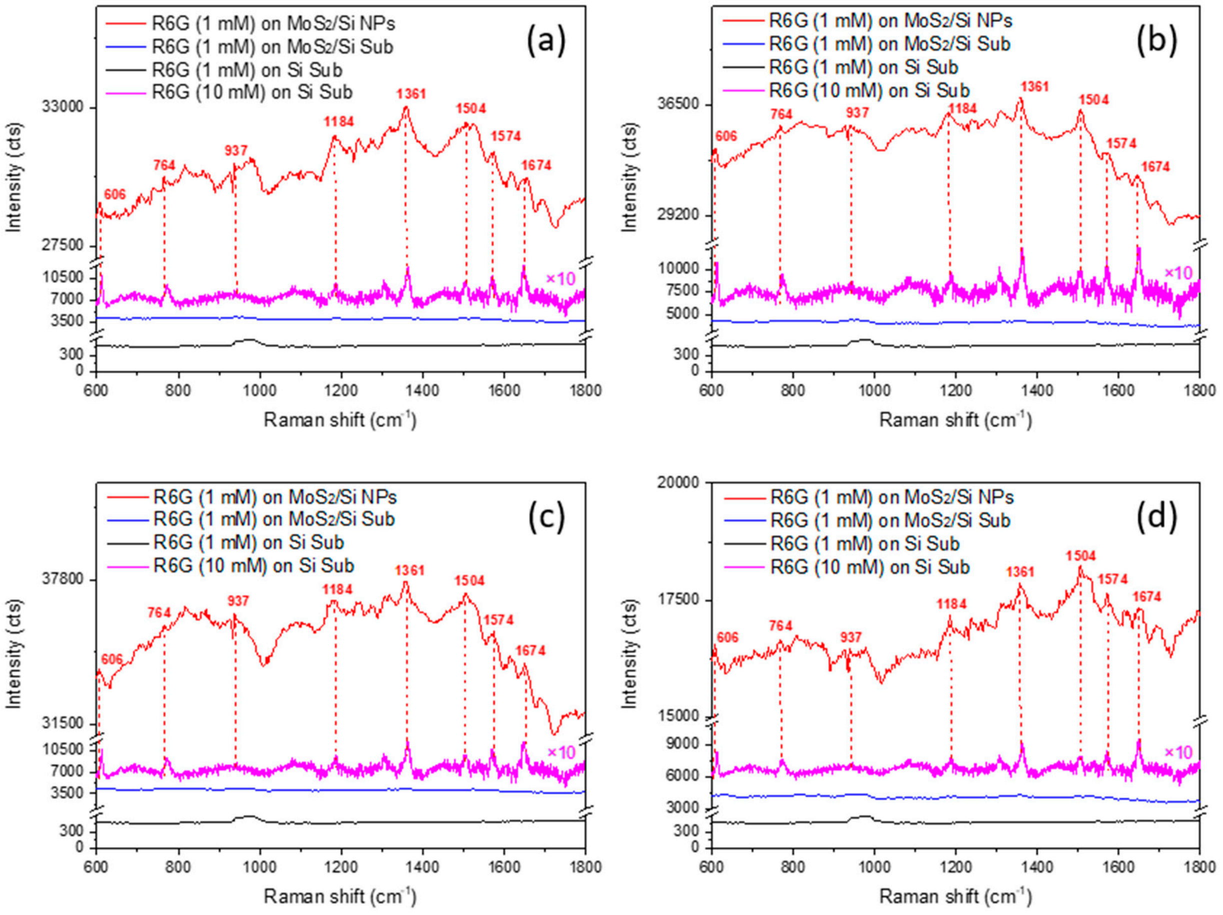

Figure S1. SERS signals of the R6G molecules on the Si, MoS_2 thin film and Si/MoS_2 core-shell NP arrays substrates. An additional SERS signal of the R6G molecules (10 mM) on a Si substrate is included for comparison. The red SERS curves in (a)~(d) were measured from the different positons of the R6G molecules on the Si/MoS_2 core-shell NP arrays substrates.
